# Tightening Monogamy and Polygamy Inequalities of Multiqubit Entanglement

**DOI:** 10.1038/s41598-018-37731-z

**Published:** 2019-03-01

**Authors:** Ahmad Farooq, Junaid ur Rehman, Youngmin Jeong, Jeong San Kim, Hyundong Shin

**Affiliations:** 1 Department of Electronic Engineering, Kyung Hee University, Yongin-si, 17104 Korea; 2 Department of Applied Mathematics and Institute of Natural Sciences, Kyung Hee University, Yongin-si, 17104 Korea

## Abstract

Monogamy and polygamy relations of quantum entanglement characterize the sharing and distribution of entanglement in a multipartite system. Multiqubit entanglement can be characterized entirely with bipartite combinations by saturating the monogamy and polygamy inequalities. In this paper, we tighten monogamy and polygamy constraints for the squared convex-roof extended negativity and its dual measure by employing a genetic algorithm. This evolutionary algorithm optimizes inequality residual functions to improve the monogamy and polygamy relations of these entanglement measures.

## Introduction

Entanglement is a quantum mechanical phenomenon enabling spatially separated parties to share quantum correlations in a manner that is not possible in classical systems^1–3^. To characterize, utilize, and quantify this unique phenomenon, entanglement measures, properties, and applications have been reported in the literature^4–8^.

One distinct property of entanglement is its limited shareability. This property is eloquently captured by the monogamy relation of entanglement^9^. The entanglement monogamy states that if two parties *A* and *B* are *maximally* entangled with each other, then they *cannot* be entangled with any third party *C*. More generally, individual bipartite quantum correlations are highly restricted by the amount of quantum correlations between *C* and *AB*. This statement can be further generalized to a multipartite scenario and similar restrictions on the amount of individual correlations can be imposed^10–13^. These monogamy relations provide a way to characterize different types of entanglement sharing. The monogamy of entanglement is also an important element in the analysis of quantum information protocols, such as quantum cryptography^14^ and quantum channel discrimination^15^. Entanglement of assistance is a notion that is dual to entanglement measures^16^. It can be viewed as the maximum amount of entanglement that the party *C* can distribute between *A* and *B* by performing measurements on his own subsystem^17^. While the quantum entanglement is monogamous, the entanglement of assistance is known to be polygamous^18^.

The concepts of monogamy and polygamy of the multipartite entangled state are concretely represented in the form of mathematical inequalities. Saturation of the monogamy inequality implies the complete characterization of multipartite entanglement^19^. On the other hand, the saturation of the polygamy inequality provides a finer characterization of the entanglement distribution^20^. Therefore, there are recent attempts to tightening these relations by raising the entanglement measures to a power and then utilizing some elementary mathematical inequalities^21–25^.

In this paper, we use a genetic algorithm (GA) to tighten the monogamy and polygamy inequalities. The GA belongs to a broad class of algorithms known as evolutionary algorithms (EAs)^26^. The EAs mimic the process of evolution in species over multiple generations^27^. Each generation consists of individuals whose fitness for an objective function is calculated. Survivals demonstrating higher fitness for the objective function are passed on to the next generation (either directly, or after crossover and mutation with other individuals), whereas the weaker individuals are removed. This process is stochastic and generally spans several generations^28^. The main advantage of the GA is that it can solve any optimization problem even if it is not convex. One problem associated with the GA is its possibility to give local minima^29^. Since the GA is easy to implement without any constraints, we use this algorithm to solve the optimization problem for tightness.

Our main focus in this paper is to tighten the monogamy and polygamy inequalities based on the squared convex-roof extended negativity (SCREN) and the SCREN of assistance (SCRENoA)^13^ for multipartite qubit systems. We first fit a shape (in terms of a mathematical expression) of the residual (the difference between both sides) of each inequality. We then optimize key parameters of the residual expression to tighten the inequality using the GA. This framework provides monogamy and polygamy inequalities that are significantly tighter than the other known bounds.

## Results

### Measures of Entanglement

Concurrence and negativity are well-known bipartite entanglement measures^4–6^. The monogamy inequality of concurrence holds true only for qubit systems while being violated for higher-dimensional (qudit) quantum systems^9,12,30^. In contrast, entanglement negativity, which is based on the positive partial transposition (PPT) criterion, holds the monogamy relation for some qudit systems as well^13^. For any bipartite quantum state *ρ*_*AB*_ with its partial transpose ${\rho }_{AB}^{{T}_{B}}$, its negativity is defined as^6^

1 $${\mathscr{N}}({\rho }_{A|B})=\parallel {\rho }_{AB}^{{T}_{B}}{\parallel }_{1}-1,$$

where $\parallel \rho {\parallel }_{1}={\rm{tr}}\sqrt{\rho {\rho }^{\dagger }}$ denotes the trace norm. Although the negativity is a computable measure of mixed-state entanglement for arbitrary dimensional quantum systems, there exist entangled states, known as PPT-bound entangled states, whose negativity is zero. For the ability to distinguish the PPT-bound entanglement from separability, an extension of negativity has been presented by the convex-roof construction^7,31^.

For a bipartite mixed state ${\rho }_{AB}={\sum }_{i}\,{p}_{i}{|{\psi }_{i}\rangle }_{AB}\langle {\psi }_{i}|$ where 0 ≤ *p*_*i*_ ≤ 1, $\forall i$, and ${\sum }_{i}\,{p}_{i}=1$, the SCREN and SCRENoA are defined respectively as^23^

2 $${{\mathfrak{N}}}_{1}\,({\rho }_{A|B})={[{\rm{\min }}\sum _{i}{p}_{i}{\mathscr{N}}({|{\psi }_{i}\rangle }_{A|B})]}^{2},$$

3 $${{\mathfrak{N}}}_{2}\,({\rho }_{A|B})={[{\rm{\max }}\sum _{i}{p}_{i}{\mathscr{N}}({|{\psi }_{i}\rangle }_{A|B})]}^{2},$$

where the minimization and maximization are over all possible pure-state decompositions of *ρ*_*AB*_. Since the SCREN and SCRENoA reduce to the squared concurrence and its dual quantity (concurrence of assistance) for any two-qubit state, these measures provide their generalization without any known examples violating their properties even in higher dimensional quantum systems^13^. Hence, the monogamy and polygamy inequalities of multiqubit entanglement are given in terms of the SCREN and SCRENoA respectively as

4 $${{\mathfrak{N}}}_{1}({|\psi \rangle }_{A|{B}_{1}\cdots {B}_{N-1}})\ge \sum _{n=1}^{N-1}\,{{\mathfrak{N}}}_{1}\,({\rho }_{A|{B}_{n}}),$$

5 $${{\mathfrak{N}}}_{2}({|\psi \rangle }_{A|{B}_{1}\cdots {B}_{N-1}})\le \sum _{n=1}^{N-1}\,{{\mathfrak{N}}}_{2}\,({\rho }_{A|{B}_{n}}),$$

for any *N*-qubit pure state ${|\psi \rangle }_{A{B}_{1}\cdots {B}_{N-1}}$ and its two-qubit reduced density matrices ${\rho }_{A{B}_{n}}$ of subsytems *AB*_*n*_, *n* = 1, 2, …, *N* − 1.

### Tightening Monogamy and Polygamy Relations

We use two inequalities in this section, which will be obtained using the GA in Sec. Methods. Two inequalities are given by

6 $${(1+x)}^{\alpha }\ge 1+f(x;\alpha ){x}^{\alpha },\,{\rm{for}}\,0\le x\le 1,\alpha \ge 1,$$

7 $${(1+x)}^{\beta }\le 1+g(x;\beta ){x}^{\beta },\,{\rm{for}}\,0\le x\le 1,0\le \beta \le 1,$$

where

8 $$f(x;\alpha )={2}^{\alpha }-1+0.0263\,{(\alpha -1)}^{5.66}\,{(1-x)}^{{\alpha }^{0.115}}{x}^{\frac{{\alpha }^{1.643}}{5.339}-\alpha },$$

9 $$g(x;\beta )={2}^{\beta }-1-{\beta }^{1.455}\,{(1-\beta )}^{1.114}\,{(1-x)}^{1.112}{x}^{{\beta }^{1.358}-\beta }.$$

#### **Theorem 1**.

*For any multipartite pure state* ${|\psi \rangle }_{{A}_{1}{A}_{2}\cdots {A}_{N}}$, *we can always have* ${|\psi \rangle }_{A{B}_{1}{B}_{2}\cdots {B}_{N-1}}$ *after ordering and reindexing of its subsystems*, *such that*

10 $${{\mathfrak{N}}}_{1}\,({\rho }_{A|{B}_{n}})\ge {{\mathfrak{N}}}_{1}\,({\rho }_{A|{B}_{n+1}})\ge 0,$$

*for n* = 1, 2, …, *N* − 2. *For any α* ≥ 1, *we have the monogamy relation as*

11 $${{\mathfrak{N}}}_{1}^{\alpha }\,({|\psi \rangle }_{A|{B}_{1}{B}_{2}\cdots {B}_{N-1}})\ge {{\mathfrak{N}}}_{1}^{\alpha }\,({\rho }_{A|{B}_{1}})+\sum _{k=2}^{N-1}\,f\,(\tfrac{{{\mathfrak{N}}}_{1}\,({\rho }_{A|{B}_{k}})}{{\sum }_{n=1}^{k-1}\,{{\mathfrak{N}}}_{1}\,({\rho }_{A|{B}_{n}})};\alpha )\,{{\mathfrak{N}}}_{1}^{\alpha }\,({\rho }_{A|{B}_{k}}).$$

#### *Proof*.

Since the SCREN is nonnegative, the monogamy inequality (4) can be rewritten as

12 $${{\mathfrak{N}}}_{1}^{\alpha }\,({|\psi \rangle }_{A|{B}_{1}{B}_{2}\cdots {B}_{N-1}})\ge {[\sum _{n=0}^{N-1}{{\mathfrak{N}}}_{1}({\rho }_{A|{B}_{n}})]}^{\alpha },$$

for any *α* ≥ 1. For a multiqubit pure state ${|\psi \rangle }_{A{B}_{1}{B}_{2}\cdots {B}_{N-1}}$ with its reduced density matrices ${\rho }_{A{B}_{n}}$ for *n* = 1, 2, …, *N* − 1, we have

13 $$\begin{array}{rcl}{[\sum _{n=1}^{N-1}{{\mathfrak{N}}}_{1}({\rho }_{A|{B}_{n}})]}^{\alpha } & = & {[\sum _{n=1}^{N-2}{{\mathfrak{N}}}_{1}({\rho }_{A|{B}_{n}})+{{\mathfrak{N}}}_{1}({\rho }_{A|{B}_{N-1}})]}^{\alpha }\\  & = & {[\sum _{n=1}^{N-2}{{\mathfrak{N}}}_{1}({\rho }_{A|{B}_{n}})]}^{\alpha }\,{[1+\tfrac{{{\mathfrak{N}}}_{1}({\rho }_{A|{B}_{N-1}})}{{\sum }_{n=1}^{N-2}{{\mathfrak{N}}}_{1}({\rho }_{A|{B}_{n}})}]}^{\alpha }\\  & \ge  & {[\sum _{n=1}^{N-2}{{\mathfrak{N}}}_{1}({\rho }_{A|{B}_{n}})]}^{\alpha }\,[1+f(\tfrac{{{\mathfrak{N}}}_{1}\,({\rho }_{A|{B}_{N-1}})}{{\sum }_{n=1}^{N-2}\,{{\mathfrak{N}}}_{1}\,({\rho }_{A|{B}_{n}})};\alpha ){(\tfrac{{{\mathfrak{N}}}_{1}({\rho }_{A|{B}_{N-1}})}{{\sum }_{n=1}^{N-2}{{\mathfrak{N}}}_{1}({\rho }_{A|{B}_{n}})})}^{\alpha }]\end{array}$$

14 $$\begin{array}{rcl} & = & {[\sum _{n=1}^{N-2}{{\mathfrak{N}}}_{1}({\rho }_{A|{B}_{n}})]}^{\alpha }+f(\tfrac{{{\mathfrak{N}}}_{1}\,({\rho }_{A|{B}_{N-1}})}{{\sum }_{n=1}^{N-2}\,{{\mathfrak{N}}}_{1}\,({\rho }_{A|{B}_{n}})};\alpha )\,{{\mathfrak{N}}}_{1}^{\alpha }\,({\rho }_{A|{B}_{N-1}})\\  & \ge  & {[\sum _{n=1}^{N-3}{{\mathfrak{N}}}_{1}({\rho }_{A|{B}_{n}})]}^{\alpha }+\sum _{k=N-2}^{N-1}\,f\,(\tfrac{{{\mathfrak{N}}}_{1}\,({\rho }_{A|{B}_{k}})}{{\sum }_{n=1}^{k-1}\,{{\mathfrak{N}}}_{1}\,({\rho }_{A|{B}_{n}})};\alpha )\,{{\mathfrak{N}}}_{1}^{\alpha }\,({\rho }_{A|{B}_{k}})\\  & \ge  & {{\mathfrak{N}}}_{1}^{\alpha }\,({\rho }_{A|{B}_{1}})+\sum _{k=2}^{N-1}\,f\,(\tfrac{{{\mathfrak{N}}}_{1}\,({\rho }_{A|{B}_{k}})}{{\sum }_{n=1}^{k-1}\,{{\mathfrak{N}}}_{1}\,({\rho }_{A|{B}_{n}})};\alpha )\,{{\mathfrak{N}}}_{1}^{\alpha }\,({\rho }_{A|{B}_{k}}),\end{array}$$

where the inequality (13) follows from (6), (10), and the fact that

15 $$0\le \frac{{{\mathfrak{N}}}_{1}\,({\rho }_{A|{B}_{N-1}})}{{\sum }_{n=1}^{N-2}\,{{\mathfrak{N}}}_{1}\,({\rho }_{A|{B}_{n}})}\le 1,$$

and the last two inequalities are obtained by induction. Hence, using (12) and (14), we complete the proof.  $\square$

#### **Remark 1**.

Since *f*(*x*; *α*) ≥ 2^*α*^ − 1 ≥ *α* ≥ 1 for 0 ≤ *x* ≤ 1, we have

16 $$\begin{array}{l}{{\mathfrak{N}}}_{1}^{\alpha }\,({\rho }_{A|{B}_{1}})+\sum _{n=2}^{N-1}\,f\,(\frac{{{\mathfrak{N}}}_{1}\,({\rho }_{A|{B}_{n}})}{{\sum }_{k=1}^{n-1}\,{{\mathfrak{N}}}_{1}\,({\rho }_{A|{B}_{k}})};\alpha )\,{{\mathfrak{N}}}_{1}^{\alpha }\,({\rho }_{A|{B}_{n}})\\ \begin{array}{lll} & \ge  & {{\mathfrak{N}}}_{1}^{\alpha }\,({\rho }_{A|{B}_{1}})+({2}^{\alpha }-1)\,\sum _{n=2}^{N-1}\,{{\mathfrak{N}}}_{1}^{\alpha }\,({\rho }_{A|{B}_{n}})\end{array}\end{array}$$

17 $$\begin{array}{l}\ge {{\mathfrak{N}}}_{1}^{\alpha }\,({\rho }_{A|{B}_{1}})+\alpha \,\sum _{n=2}^{N-1}\,{{\mathfrak{N}}}_{1}^{\alpha }\,({\rho }_{A|{B}_{n}})\end{array}$$

18 $$\begin{array}{l}\ge \sum _{n=1}^{N-1}\,{{\mathfrak{N}}}_{1}^{\alpha }\,({\rho }_{A|{B}_{n}})\end{array}$$

where the bounds (16–18) are used in the monogamy relations^13,23,24^. Hence, Theorem 1 provides a tighter inequality than these known bounds.

#### **Remark 2**.

Theorem 1 also holds true for any *N*-qubit mixed state ${\rho }_{A{B}_{1}\cdots {B}_{N-1}}$ due to the inequality^32^

19 $${{\mathfrak{N}}}_{1}\,({\rho }_{A|{B}_{1}\cdots {B}_{N-1}})\ge \sum _{n=1}^{N-1}\,{{\mathfrak{N}}}_{1}\,({\rho }_{A|{B}_{n}}).$$

Using the same arguments in Theorem 1, we get the tight monogamy inequality for multipartite mixed states as follows:

20 $${{\mathfrak{N}}}_{1}^{\alpha }\,({\rho }_{A|{B}_{1}{B}_{2}\cdots {B}_{N-1}})\ge {{\mathfrak{N}}}_{1}^{\alpha }\,({\rho }_{A|{B}_{1}})+\sum _{k=2}^{N-1}\,f\,(\tfrac{{{\mathfrak{N}}}_{1}({\rho }_{A|{B}_{k}})}{{\sum }_{n=1}^{k-1}\,{{\mathfrak{N}}}_{1}\,({\rho }_{A|{B}_{n}})};\alpha )\,{{\mathfrak{N}}}_{1}^{\alpha }\,({\rho }_{A|{B}_{k}}).$$

#### **Theorem 2**.

*For any multipartite pure state* ${|\psi \rangle }_{{A}_{1}{A}_{2}\cdots {A}_{N}}$, *we can always have* ${|\psi \rangle }_{A{B}_{1}{B}_{2}\cdots {B}_{N-1}}$ *after ordering and reindexing of its subsystems*, *such that*

21 $${{\mathfrak{N}}}_{2}\,({\rho }_{A|{B}_{n}})\ge {{\mathfrak{N}}}_{2}\,({\rho }_{A|{B}_{n+1}})\ge 0,$$

*for n* = 1, 2, …, *N* − 2. *For any* 0 ≤ *β* ≤ 1, *we have the polygamy relation as*

22 $${{\mathfrak{N}}}_{2}^{\beta }\,({|\psi \rangle }_{A|{B}_{1}{B}_{2}\cdots {B}_{N-1}})\le {{\mathfrak{N}}}_{2}^{\beta }\,({\rho }_{A|{B}_{1}})+\sum _{n=2}^{N-1}\,g\,(\tfrac{{{\mathfrak{N}}}_{2}\,({\rho }_{A|{B}_{n}})}{\sum _{k=1}^{n-1}\,{{\mathfrak{N}}}_{2}\,({\rho }_{A|{B}_{k}})};\beta )\,{{\mathfrak{N}}}_{2}^{\beta }\,({\rho }_{A|{B}_{n}}).$$

#### *Proof*.

Since the SCRENoA is nonnegative, the polygamy inequality (5) can be rewritten as

23 $${{\mathfrak{N}}}_{2}^{\beta }\,({|\psi \rangle }_{A|{B}_{1}\cdots {B}_{N-1}})\le {[\sum _{n=1}^{N-1}{{\mathfrak{N}}}_{2}({\rho }_{A|{B}_{n}})]}^{\beta },$$

for 0 ≤ *β* ≤ 1. Using the same arguments in the proof of Theorem 1, we get

24 $$\begin{array}{l}{{\mathfrak{N}}}_{2}^{\beta }\,({|\psi \rangle }_{A|{B}_{1}\cdots {B}_{N-1}})\\ \begin{array}{rcl} & \le  & {[\sum _{n=1}^{N-2}{{\mathfrak{N}}}_{2}({\rho }_{A|{B}_{n}})]}^{\beta }{[1+\tfrac{{{\mathfrak{N}}}_{2}({\rho }_{A|{B}_{N-1}})}{{\sum }_{n=1}^{N-2}{{\mathfrak{N}}}_{2}({\rho }_{A|{B}_{n}})}]}^{\beta }\\  & \le  & {[\sum _{n=1}^{N-2}{{\mathfrak{N}}}_{2}({\rho }_{A|{B}_{n}})]}^{\beta }+g(\tfrac{{{\mathfrak{N}}}_{2}\,({\rho }_{A|{B}_{N-1}})}{{\sum }_{n=1}^{N-2}\,{{\mathfrak{N}}}_{2}\,({\rho }_{A|{B}_{n}})};\beta )\,{{\mathfrak{N}}}_{2}^{\beta }\,({\rho }_{A|{B}_{N-1}})\\  & \le  & {[\sum _{n=1}^{N-3}{{\mathfrak{N}}}_{2}({\rho }_{A|{B}_{n}})]}^{\beta }+\sum _{k=N-2}^{N-1}\,g\,(\tfrac{{{\mathfrak{N}}}_{2}\,({\rho }_{A|{B}_{k}})}{{\sum }_{n=1}^{k-1}\,{{\mathfrak{N}}}_{2}\,({\rho }_{A|{B}_{n}})};\beta )\,{{\mathfrak{N}}}_{2}^{\beta }\,({\rho }_{A|{B}_{k}})\\  & \le  & {{\mathfrak{N}}}_{2}^{\beta }\,({\rho }_{A|{B}_{1}})+\sum _{k=2}^{N-2}\,g\,(\tfrac{{{\mathfrak{N}}}_{2}\,({\rho }_{A|{B}_{k}})}{{\sum }_{n=1}^{k-1}\,{{\mathfrak{N}}}_{2}\,({\rho }_{A|{B}_{n}})};\beta )\,{{\mathfrak{N}}}_{2}^{\beta }\,({\rho }_{A|{B}_{k}}),\end{array}\end{array}$$

which complete the proof.$\square$

#### **Remark 3**.

Since *g*(*x*; *β*) ≤ 2^*β*^ − 1 ≤ *β* ≤ 1 for 0 ≤ *β* ≤ 1, we have

25 $$\begin{array}{c}{{\mathfrak{N}}}_{2}^{\beta }\,({\rho }_{A|{B}_{1}})+\sum _{n=2}^{N-1}\,g\,(\frac{{{\mathfrak{N}}}_{2}\,({\rho }_{A|{B}_{n}})}{{\sum }_{k=1}^{n-1}\,{{\mathfrak{N}}}_{2}\,({\rho }_{A|{B}_{k}})};\beta )\,{{\mathfrak{N}}}_{2}^{\beta }\,({\rho }_{A|{B}_{n}})\\ \,\le \,{{\mathfrak{N}}}_{2}^{\beta }\,({\rho }_{A|{B}_{1}})+\beta \,\sum _{n=2}^{N-1}\,{{\mathfrak{N}}}_{2}^{\beta }\,({\rho }_{A|{B}_{n}})\end{array}$$

26 $$\,\le \,\sum _{n=1}^{N-1}\,{{\mathfrak{N}}}_{2}^{\beta }\,({\rho }_{A|{B}_{n}})$$

where the bounds (25) and (26) are used in the polygamy relations^13,23^. Hence, Theorem 2 provides a tighter polygamy inequality than these known bounds.

## Discussion

For a numerical example, we consider a generalized tripartite qubit system^33^

27 $${|\psi \rangle }_{{A}_{1}{A}_{2}{A}_{3}}={\mu }_{0}|000\rangle +{\mu }_{1}{e}^{\iota \phi }|100\rangle +{\mu }_{2}|101\rangle +{\mu }_{3}|110\rangle +{\mu }_{4}|111\rangle ,$$

where *μ*_*i*_ ≥ 0, $\forall i$, and ${\sum }_{i=0}^{4}\,{\mu }_{i}^{2}=1$. For this tripartite qubit system, the SCREN and SCRENoA are computed as

28 $$\{\begin{array}{rcl}{{\mathfrak{N}}}_{1}\,({|\psi \rangle }_{{A}_{1}|{A}_{2}{A}_{3}}) & = & 4{\mu }_{0}^{2}({\mu }_{2}^{2}+{\mu }_{3}^{2}+{\mu }_{4}^{2}),\\ {{\mathfrak{N}}}_{1}\,({\rho }_{{A}_{1}|{A}_{2}}) & = & 4{\mu }_{0}^{2}{\mu }_{3}^{2},\\ {{\mathfrak{N}}}_{1}\,({\rho }_{{A}_{1}|{A}_{3}}) & = & 4{\mu }_{0}^{2}{\mu }_{2}^{2},\end{array}$$

29 $$\{\begin{array}{rcl}{{\mathfrak{N}}}_{2}\,({|\psi \rangle }_{{A}_{1}|{A}_{2}{A}_{3}}) & = & 4{\mu }_{0}^{2}\,({\mu }_{2}^{2}+{\mu }_{3}^{2}+{\mu }_{4}^{2}),\\ {{\mathfrak{N}}}_{2}\,({\rho }_{{A}_{1}|{A}_{2}}) & = & 4{\mu }_{0}^{2}\,({\mu }_{3}^{2}+{\mu }_{4}^{2}),\\ {{\mathfrak{N}}}_{2}\,({\rho }_{{A}_{1}|{A}_{3}}) & = & 4{\mu }_{0}^{2}\,({\mu }_{2}^{2}+{\mu }_{4}^{2}).\end{array}$$

**Figure 1:**
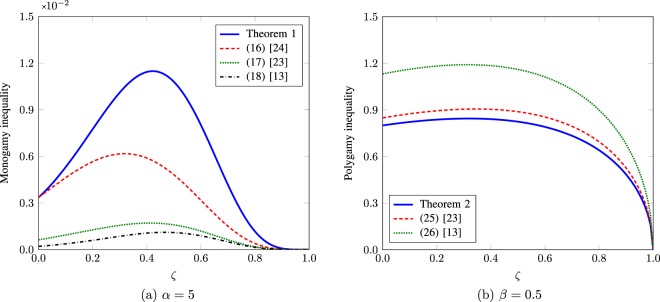
For the tripartite qubit system (27) with ${\mu }_{i}^{2}={\lambda }_{i}$, *i* = 0, 1, 2, 3, 4, where *λ*_*i*_’s are the decreasing-ordered eigenvalues of *R*; (**a**) the monogamy inequality (11) in Theorem 1 when *α* = 5 and (**b**) the polygamy inequality (22) in Theorem 2 when *β* = 0.5 as a function of *ζ*. For comparison, we also plot the known bounds (16–18), (25), and (26) for the monogamy and polygamy relations. We can see that our monogamy and polygamy inequalities in Theorems 1 and 2 are tighter than these known bounds.

To demonstrate the tightness of monogamy and polygamy inequalities in Theorems 1 and 2, we distribute *μ*_*i*_’s in (27) using the eigenvalues of the following exponential correlation matrix^34^

30 $$R=\frac{1}{5}\,(\begin{array}{ccccc}1 & \zeta  & {\zeta }^{2} & {\zeta }^{3} & {\zeta }^{4}\\ \zeta  & 1 & \zeta  & {\zeta }^{2} & {\zeta }^{3}\\ {\zeta }^{2} & \zeta  & 1 & \zeta  & {\zeta }^{2}\\ {\zeta }^{3} & {\zeta }^{2} & \zeta  & 1 & \zeta \\ {\zeta }^{4} & {\zeta }^{3} & {\zeta }^{2} & \zeta  & 1\end{array}),$$

where $\zeta \in [0,1]$ is a correlation coefficient. Let *λ*_0_ ≥ *λ*_1_ ≥ *λ*_2_ ≥ *λ*_3_ ≥ *λ*_4_ ≥ 0 be the eigenvalues of *R* in decreasing order and set ${\mu }_{i}^{2}={\lambda }_{i}$, $i=0,1,2,3,4$. Since tr(*R*) = 1, we have ${\sum }_{i=0}^{4}\,{\mu }_{i}^{2}=1$. Using (28) and (29), we plot the monogamy and polygamy inequalities in Theorems 1 and 2 as a function of *ζ* for the tripartite qubit system (27) in Fig. 1 when (a) *α* = 5 and (b) *β* = 0.5, respectively. The known bounds (16–18), (25), and (26) for the monogamy and polygamy relations are also depicted for comparison.

We can see from (28) and (29) that a tripartite state with *μ*_4_ = 0, e.g., the *W*-class state (*μ*_1_ = *μ*_4_ = 0), saturates the monogamy and polygamy inequalities, while a tripartite state with *μ*_2_ = *μ*_3_ = 0, e.g., the GHZ-class state (*μ*_1_ = *μ*_2_ = *μ*_3_ = 0), yields the maximum residuals of monogamy and polygamy inequalities. Tightening the monogamy and polygamy inequalities enables us to precisely characterize the entanglement sharing and distribution in a multipartite scenario. Our framework can also be used in other entanglement measures such as the entanglement of formation, Tsallis entropy, Rényi entropy, and unified entropy for qubit systems.

## Methods

In this section, we first tighten a known inequality, which is used for obtaining tight monogamy relations, by identifying a mathematical expression for the residual of this inequality and fine-tuning its parameters by the GA. Next, we derive an inequality, which is tighter than the known results for polygamy, and then further tighten this inequality by using again the GA.

Tightening the inequality by fitting a parametric form of its residual is a nonlinear optimization problem. We can employ the GA to perform this optimization due to its ability to handle discontinuous, nonlinear, and nondifferentiable objective functions^26–29^. Specifically, the GA randomly generates candidate solutions within given constraints and mimics the evolution process (the survival of the fittest) in searching the optimal solution. The promising candidates from one generation are identified and utilized to produce the next generation of candidate solutions. This process is iterated over several generations until a stopping condition is satisfied. A stochastic search of the GA sometimes leads to local optima that can steer the search in a wrong direction. To overcome this problem, we can work on the population size, mutation rate, crossover probability, and termination condition^26–29^. In our optimization problem, we increase the population size more than 10,000, set the termination condition to be strictly 10^−30^, and restrict the lower limit of parameter variables to be nonnegative by looking at the landscape of our fitness function.

**Figure 2:**
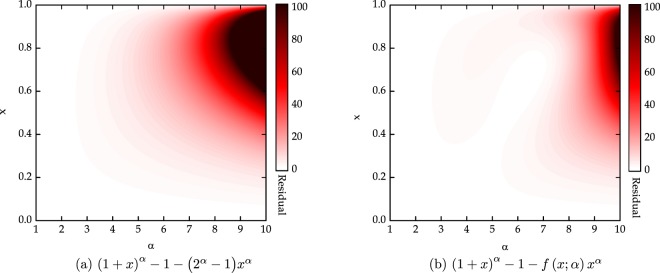
Residuals of (**a**) the inequality (31) and (**b**) the inequality (6) as a function of (*x*, *α*). We can see that the inequality (6) obtained by optimizing the parameters with the GA is significantly tighter than the known inequality (31).

We begin with the known inequality^22^

31 $${(1+x)}^{\alpha }\ge 1+({2}^{\alpha }-1){x}^{\alpha },$$

where 0 ≤ *x* ≤ 1 and *α* ≥ 1. The inequality residual (1 + *x*)^*α*^ − 1 − (2^*α*^ − 1)*x*^*α*^ is plotted as a function of (*x*, *α*) in Fig. 2(a). We identify this curve to be of the form

32 $$p(x;\alpha ,{\bf{u}})={u}_{1}{(\alpha -1)}^{{u}_{2}}\,{(1-x)}^{{\alpha }^{{u}_{3}}}{x}^{\frac{{\alpha }^{{u}_{4}}}{{u}_{5}}},$$

where **u** = [*u*_1_, *u*_2_, *u*_3_, *u*_4_, *u*_5_] is a parameter vector to be optimized for tightening the inequality; *u*_1_ is a scaling parameter; and *u*_2_, *u*_3_, *u*_4_, and *u*_5_ are shape parameters. Now, we can formulate our optimization problem for 0 ≤ *x* ≤ 1 and *α* ≥ 1 as follows:

33 $$\begin{array}{l}\mathop{{\rm{\max }}}\limits_{{\bf{u}}}\,p\,(x;\alpha ,{\bf{u}})\\ {\rm{subject}}\,\mathrm{to}:\,{(1+x)}^{\alpha }\ge 1+({2}^{\alpha }-1){x}^{\alpha }+p(x;\alpha ,{\bf{u}}).\end{array}$$

By solving this optimization problem with the GA, we find the best parameter vector

34 $${\bf{u}}=[0.0263,5.66,0.155,1.643,5.339],$$

leading to a tigher inequality (6). The residual (1 + *x*)^*α*^ − 1 − *f*(*x*; *α*)*x*^*α*^ of the inequality (6) is plotted as a function of (*x*, *α*) in Fig. 2(b). It can be seen from Fig. 2 that this optimized inequality (6) is significantly tighter than the inequality (31).

For the polygamy inequality, we first derive an opposite-side inequality and then take the same steps to tighten it using the GA.

### **Lemma 1**.

*For* 0 ≤ *x* ≤ 1 *and* 0 ≤ *β* ≤ 1, *we have*

35 $${(1+x)}^{\beta }\le 1+({2}^{\beta }-1){x}^{\beta }.$$

### *Proof*.

Let *h*(*y*; *β*) = (1 + *y*)^*β*^ − *y*^*β*^. Then, for *y* ≥ 1 and 0 ≤ *β* ≤ 1, we have

36 $$\frac{dh(y;\beta )}{dy}=\beta [{(1+y)}^{\beta -1}-{y}^{\beta -1}]\le 0,$$

which implies that the function is decreasing in *y* ≥ 1. Hence,

37 $${(1+y)}^{\beta }-{y}^{\beta }\le h(1;\beta )={2}^{\beta }-1,$$

for *y* ≥ 1. Plugging *y* = 1/*x* in (37), we complete the proof.$\square$

**Figure 3:**
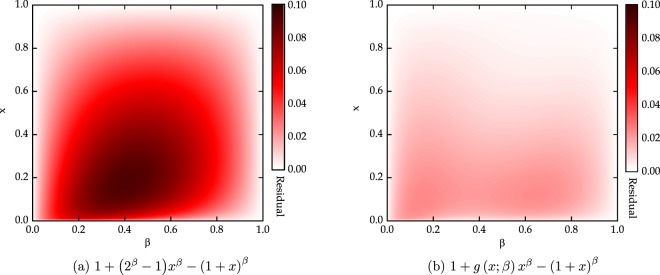
Residuals of (**a**) the inequality (35) and (**b**) the inequality (7) as a function of (*x*, *β*). It can be seen that the inequality (7) optimized by the GA is significantly tighter than the inequality (35).

The residual 1 + (2^*β*^ − 1) − *x*^*β*^(1 + *x*)^*β*^ is plotted as a function of (*x*, *β*) in Fig. 3(a) and this curve is fitted to the following expression

38 $$q(x;\beta ,{\bf{v}})={\beta }^{{v}_{1}}{(1-\beta )}^{{v}_{2}}{(1-x)}^{{v}_{3}}{x}^{{\beta }^{{v}_{4}}},$$

where **v** = [*v*_1_, *v*_2_, *v*_3_, *v*_4_] is a parameter vector to be optimized for tightening the inequality. To further tighten the inequality (35), we now formulate an optimization problem for 0 ≤ *x* ≤ 1 and 0 ≤ *β* ≤ 1 as follows:

39 $$\begin{array}{l}\mathop{{\rm{\max }}}\limits_{{\bf{v}}}\,q\,(x;\beta ,{\bf{v}})\\ {\rm{subject}}\,\mathrm{to}:\,{(1+x)}^{\beta }\le 1+({2}^{\beta }-1){x}^{\beta }-q(x;\beta ,{\bf{v}}).\end{array}$$

Using the GA, we obtain the parameter vector

40 $${\bf{v}}=[1.455,1.114,1.112,1.358],$$

leading to a tigher inequality (7). The residual 1 + *g*(*x*; *β*)*x*^*β*^ − (1 + *x*)^*β*^ of the inequality (7) is plotted as a function of (*x*, *β*) in Fig. 3(b) for comparison of tightness with the inequality (35).
